# Supplementary material for: Crystal Structure of Staphopain C from Staphylococcus aureus
Source: Molecules. 2023 May 29;28(11):4407. doi: 10.3390/molecules28114407 (PMC10254297; doi:10.3390/molecules28114407)
Supplement: Supplementary file 1 [file molecules-28-04407-s001.zip › molecules-2344295-supplementary.pdf]

Supporting information

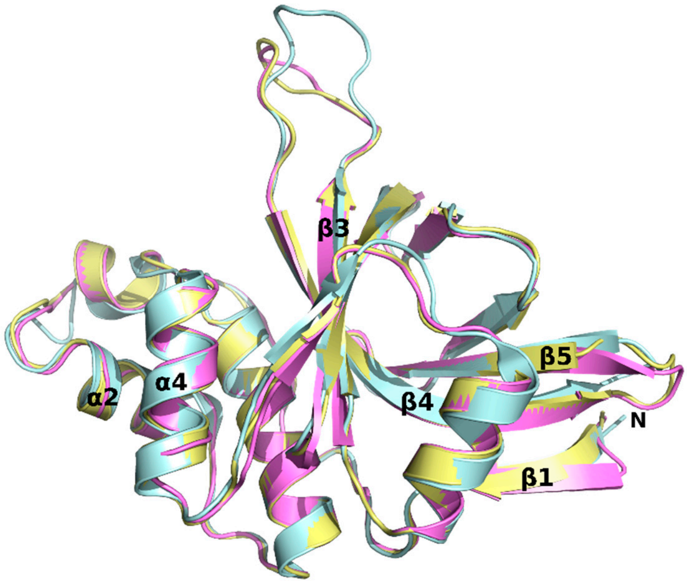

Figure S1. Overlay of reported X-ray structures of staphopains from *Staphylococcus aureus*: 8OIG (staphopain C - this study) - purple, 1CV8 (staphopain A) - yellow, 1Y4H (staphopain B) – light blue.

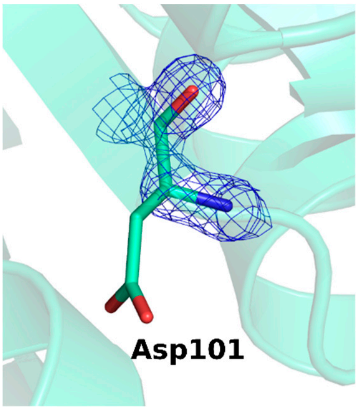

Figure S2. The 2|Fo|- |Fc| electron density map at 1.58 Å resolution is contoured at 1.6 σ around the Asp101 residue.

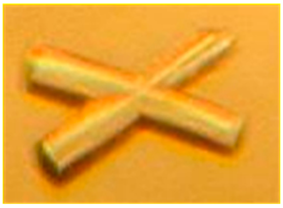

Figure S3. X-shaped crystal of ScpA2 protein.
